# Leveraging machine learning to examine engagement with a digital therapeutic

**DOI:** 10.3389/fdgth.2023.1063165

**Published:** 2023-06-02

**Authors:** Andrew C. Heusser, Denton J. DeLoss, Elena Cañadas, Titiimaea Alailima

**Affiliations:** Akili Interactive, Boston, MA, United States

**Keywords:** digital therapeutics, engagement, machine learning, cognition, brain health

## Abstract

Digital Therapeutics (DTx) are evidence-based software-driven interventions for the prevention, management, and treatment of medical disorders or diseases. DTx offer the unique ability to capture rich objective data about when and how a patient engages with a treatment. Not only can one measure the quantity of patient interactions with a digital treatment with high temporal precision, but one can also assess the quality of these interactions. This is particularly useful for treatments such as cognitive interventions, where the specific manner in which a patient engages may impact likelihood of treatment success. Here, we present a technique for measuring the quality of user interactions with a digital treatment in near-real time. This approach produces evaluations at the level of a roughly four-minute gameplay session (mission). Each mission required users to engage in adaptive and personalized multitasking training. The training included simultaneous presentation of a sensory-motor navigation task and a perceptual discrimination task. We trained a machine learning model to classify user interactions with the digital treatment to determine if they were “using it as intended” or “not using it as intended” based on labeled data created by subject matter experts (SME). On a held-out test set, the classifier was able to reliably predict the SME-derived labels (Accuracy = .94; F1 Score = .94). We discuss the value of this approach and highlight exciting future directions for shared decision-making and communication between caregivers, patients and healthcare providers. Additionally, the output of this technique can be useful for clinical trials and personalized intervention.

## Introduction

Digital mental health interventions target the prevention or treatment of mental health disorders and associated impairments (i.e., functional, affective, cognitive) delivered via a digital platform (e.g., web browser, mobile apps, text messaging, or virtual reality) (1). They offer the potential to overcome availability and accessibility limitations, including geographical location and time (2–4).

While there are thousands of digital interventions claiming to improve various aspects of mental health, many of them have never gone through clinical trials or regulatory scrutiny. Also, due to a number of factors, including fast growth of the industry and an absence of well-accepted standards, there are widely varying definitions of what constitutes a “good” DTx (5). Contrary to wellness apps, DTx products are typically validated in rigorous clinical trials measuring safety and efficacy as well as evidence from real-world outcomes, whereas there is no such standard for wellness products (5).

Similar to traditional behavioral interventions (e.g., Cognitive Behavioral Therapy), the success of a DTx depends largely on a user's engagement (6). Broadly speaking, engagement can be described as “(a) the extent (e.g., amount, frequency, duration, depth) of usage and (b) a subjective experience characterized by attention, interest, and affect” (7). Engagement is considered to be a dynamic process that is expected to vary both within and across individuals over time (7). While data for traditional behavioral interventions is typically limited to attendance/adherence, a DTx affords the opportunity to collect rich data on when and importantly how a user interacts with the intervention.

Stakeholders across academic and industry settings acknowledge that the current measures of engagement (e.g., extent of usage) may not be sufficient (1) especially if they are not strong mediators of outcomes (8). For example, users might come back to the app every day for months (strong retention), but their symptoms do not improve. This could be interpreted as the intervention not being effective, but adherence/retention alone does not ensure that the DTx is being used as intended. The user may not have followed the instructions for use correctly, or may not have put forth significant cognitive effort and/or were distracted during use of the DTx. Another example of why standard adherence/retention methods may not be sufficient is that a user may abandon a treatment once they have achieved the desired benefits. Standard methods would predict attenuated efficacy, whereas methods focused on the quality of engagement could tell a different story. Due to their ability to collect rich data not just when but *how* a user interacts with a treatment, DTx products afford the unique opportunity to identify when a DTx product is being used as intended. Thus, an approach that provides a clinically-informed and data-driven way to measure the quality of DTx engagements may shed some light on the effectiveness of a DTx (8).

While measuring adherence and retention can be achieved by simply tracking the number of user interactions over time, assessing the quality of interactions is much more nuanced and time-consuming, and requires the expertise of trained clinicians or individuals deeply familiar with the intervention. Machine learning enables us to capture the wisdom of such experts into a classification algorithm, making this task efficient and scalable. In other words, once the classifier is trained it can be applied to large quantities of new data without the need for additional human labeling.

This manuscript introduces a machine learning-based approach to examine engagement with a pair of related DTx targeting attentional control function. These devices use proprietary algorithms designed to improve cognitive interference management in an adaptive manner and thereby personalized to the patient. Interference is instantiated through a video game-like interface presenting two tasks that are performed simultaneously (multitasking): a perceptual discrimination task (selecting the correct target from a number of distractor stimuli) and a sensory motor navigation task (continuously adjusting their position to steer towards some objects and away from others). Performance in each task is assessed during single and multitasking conditions. The interference training is adapted in real time based on the individual's performance. Thus the training is tailored specifically to each individual's performance level to achieve a consistent and optimal challenge at a predefined level of difficulty, continually challenging them to improve while providing rewards and positive feedback when they succeed.

We propose to evaluate engagement not only by examining simple adherence metrics of (e.g., sessions or total time played) but also the *quality* of the interactions with the DTx. In other words, is the user engaging with the DTx as intended (i.e., following the instructions provided, putting in an appropriate level of effort)?

## Methods & results

### Visualizing and labeling gameplay data

As described in the introduction, users engage in a perceptual discrimination and a sensory-motor task simultaneously for approximately 4 min per “mission”. The perceptual discrimination task is performed by tapping on the screen of the device while the sensory-motor task is performed by tilting the device left and right. For a video example of gameplay, see here. Missions are the basic unit of interaction with our DTx. To develop and assess the approach we used gameplay data from 1,308 missions sampled from 427 users, including users from four studies from which data has been previously published (9–12) and users of the commercial product. We pseudo-randomly sampled missions with the goal of balancing across data source (clinical or commercial), the 4 sequentially played worlds in the game, balancing types of missions (training or assessment) proportional to how frequently they occurred in the game, and selecting a variety of performance levels. The particular software build varied across studies with some differences in game content between builds, but the tasks were substantially identical and all task difficulties were governed by our proprietary cognitive training technology, the Selective-Stimulus Management Engine (SSME™). Details on the particular instantiation of SSME for a given study can be found in the papers referenced above. The data was sourced from a number of studies across a number of indications (ADHD, Multiple Sclerosis, Major Depressive Disorder) so that our classifier could learn patterns that are not indication-specific. For the clinical trials, consent for health research and publication was provided by caregivers in the form of IRB consent (please see individual studies for details). For the commercial data, retrospective IRB-exempt status was granted under 45 CFR 46 116(f)[2018 Requirements] 45 CFR 46.116(d) [Pre-2018 Requirements] for the analysis of de-identified data by the WCG Institutional Review Board on April 21, 2023 (Study Number: 1353416).

**Figure 1:**
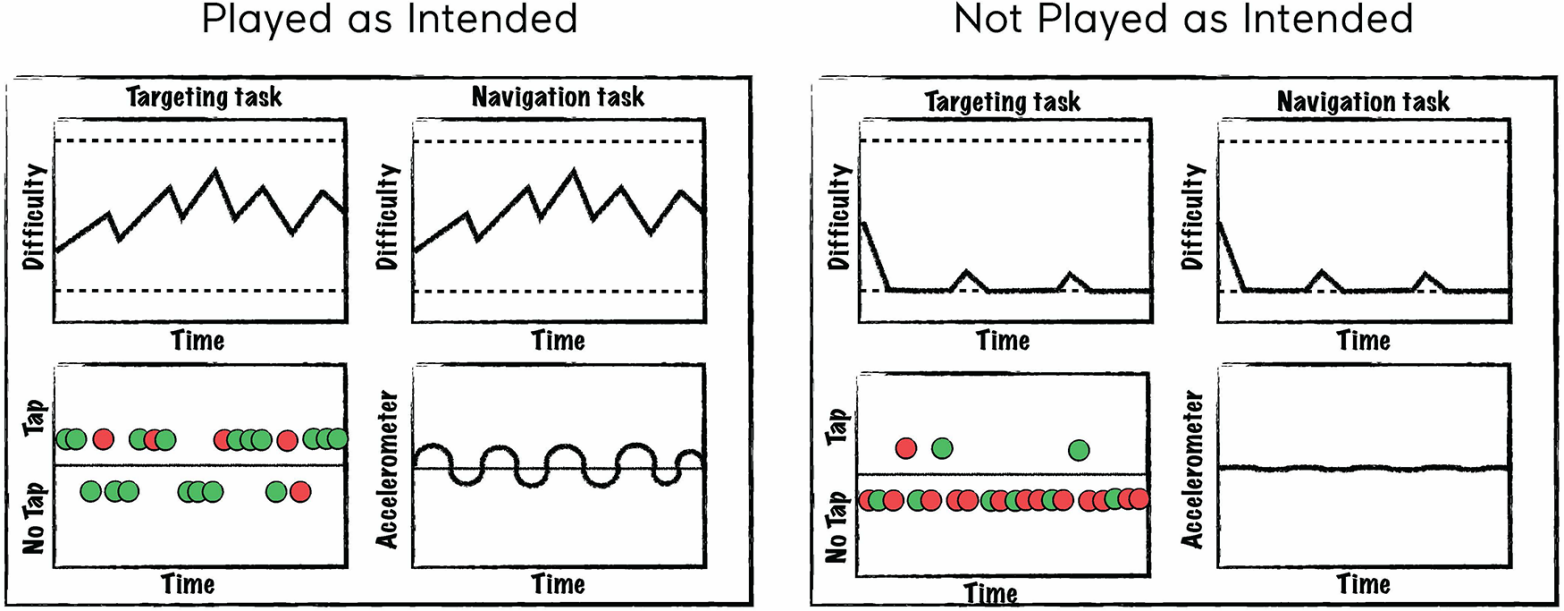
Schematic of plots created to assist in labeling. A schematic of a mission “played as intended” is represented in the left panel and “not played as intended” in the right panel. The top two plots depict difficulty levels for each of two tasks changing over time. The bottom left plot represents tapping behavior where colors indicate whether the trial was correct (green) or incorrect (red) and points above the line represent taps and points below the line represent no taps. The bottom right plot represents accelerometer input where deflections from the 0 axis indicate the degree to which the device was tilted (which controls steering in the sensory-motor navigation task).

To facilitate label generation, we created a set of plots that depict how a user is interacting with the treatment and how the treatment is dynamically responding to the user's input. The plots are generated from telemetry data that is captured as a participant engages in a mission. Figure 1 is a schematic representing a mission “played as intended” (left panel) and “not played as intended” (right panel). For each panel, the top two plots represent game difficulty levels (solid lines) that varied dynamically between the top and bottom difficulty limits (dashed lines) for each of two tasks played simultaneously. The bottom left plot represents screen tapping in response to the targeting task (green = correct tap, red = incorrect tap) and the bottom right plot represents accelerometer measurements. As seen in the left panel, “playing as intended” is characterized by dynamic changes in task difficulty as the user engages with the tasks, tapping during a reasonable percentage of targeting trials with a reasonable correct rate, and continuously varying accelerometer input representing movement of the device to perform the navigation task. In contrast, the right panel depicts a mission “not played as intended”, which is often characterized by task difficulty levels at the lower difficulty limit, infrequent taps and excessive errors, and little to no accelerometer input.

### Labeling the plots of mission data

We trained human labelers to analyze the data presented in these plots (Figure 1) and label the missions using an agreed upon strategy. The labelers included Akili employees from various departments such as Cognitive Science, Clinical Operations, and Data Science. Before they labeled the data, they were trained by reviewing a number of plots representing various examples of gameplay (e.g., playing effortfully with the correct rules for the entire mission or playing one or both tasks with the rules systematically wrong). A labeling application was created to allow labelers to indicate the proportion of time (e.g., 0, 25, 50, 75, or 100%) during each mission where each task was “played as intended”, as well as check a series of boxes if certain conditions were met (for example, if it appears they did not understand the targeting rules). These labels were only used in cases where there was reasonable certainty and typically result in accuracy levels that are far below what are in the typical range for missions. For each mission, labels were collected from 3 human labelers to increase accuracy/reduce human error. Numeric labels were transformed to binary ones for the purposes of training a binary classifier using the following operational definition for “playing as intended”: multitasking for greater than 75% of a mission while playing with the correct rules. Above this threshold is considered “playing as intended” and below the threshold is considered “not playing as intended”. We considered a full consensus from all raters of requiring 100% to be too stringent (and would lead to many false negatives) and that >50% was too liberal, which left us with >75% as the best option. A final label was determined based on the majority of the labels for each mission. For example, if 2 out of 3 labelers coded the mission as “playing as intended”, the final label was “playing as intended”.

### Model features

Features were created based on aspects of the mission data that were informative in making a decision on whether the mission was “played as intended”. To create features, we extracted the raw gameplay telemetry data that is captured for each mission played and transformed the data into a set of summary statistics. The feature set included statistics such as task accuracy, tapping frequency and accelerometer variance. These feature vectors were paired with the labels described above and were used to train a machine learning model to predict the most likely label (“played as intended” or “not played as intended”).

### Model fitting

The labeled dataset was split into a training (80%) and test (20%) set using a stratified random sampling approach (stratified by data source and label (0 or 1). A grid search was performed on the training data over hyperparameters of a Random Forest Classifier (implemented with scikit-learn version 0.23.2) using leave-one-user-out cross validation. Using the hyperparameter combination with the highest cross-validated F1-score, the random forest was retrained on the full training set. To ensure that the classifier accuracy was not inflated due to overlap in users in the training and test set, we separately analyzed the accuracy for users' data who were only in the test set (F1-score = .96) and found them to be comparable to users in both the training and test sets (F1-score = .94).

### Model results

**Figure 2:**
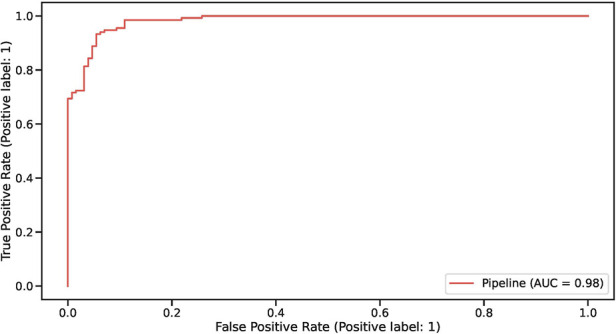
ROC curve representing model performance on the held out test set.

We validated the model by assessing performance on the held out test set. Overall, test set accuracy was 94%. An ROC curve analysis representing the model's true/false positive rate at different thresholds is shown in Figure 2. Precision, Recall and F1-score for both “playing as intended” (1) and “not playing as intended” (0) were all exactly 94% (see Table 1 for positive label metrics).

**Table 1:** Model performance statistics: accuracy, precision, recall, F1-score and support.

Accuracy	Precision	Recall	F1-score	# of Samples (positive/negative)
.94	.94	.94	.94	134/128

In addition to the model validation outlined above, we ran additional validation on new data (*n* = 600 missions) from a different set of users (*n* = 220). The labeling procedure was identical to what is described above except that there were only 2 labelers. This data was used for the purposes of model drift monitoring (i.e., the model was not retrained with this data).

Any disagreement between the labelers (“playing as intended” vs. “not playing as intended”) were reviewed together live until a consensus was reached. The F1-score for this additional validation step was similar (.92), providing additional support that the model performance was high and that model drift was unlikely to be of concern.

## Discussion

In this manuscript, we introduce a machine learning-based approach to examining engagement with a DTx targeting attentional control function. Our results suggest that it is feasible to label missions (the “units” of interaction with our DTx) based on whether or not they are “played as intended” with high accuracy. Importantly, this labeling can be done in an automated and scalable way (without continual expert assessment), which opens the door for many potential use cases centered around measuring the quality of engagement with a DTx.

A recent opinion piece (1) calls for better measures of attrition and engagement. The approach described herein fills that unmet need in the DTx space, opening a new dimension for assessing engagement. It can also help to tease apart whether attrition is due to lack of use, or due to the manner of use, and improve the product experience accordingly. The proposed approach helps the DTx be more accurate and proactive in determining whether the patient is engaging with the product as intended and can help address many of the issues brought up in the opinion piece. The authors mention gamification of the DTx as means to increase engagement, which is core to the DTx under examination. The interactivity of the game experience produces a rich data stream that enables an approach like this to be developed. But the output of this approach can enable further gamification, such as points for completing your daily tasks in the intended manner, or simple rewards for periods of significant effort when the DTx is used as intended. The approach we describe also enables more direct feedback to the patient and/or their caregivers in a near real-time manner as to whether they were using the DTx as intended, potentially paired with further messaging to encourage proper engagement to maximize benefit. These messages should be tailored to each app and given in a positive/motivational manner, to avoid inducing frustration or dissatisfaction with the DTx for the patient.

This kind of approach has several other potential uses. It could also be used to discover cohorts of patients for any number of analyses. These cohorts could be used to identify responders, examine dosage at a much finer level, or even to predict whether a user is likely to cease using the product altogether. Different cohorts may require different types of messaging to the patient depending on their usage patterns. The approach could also provide patients or their caregivers additional insights on (a) the time course of engagement over the course of a treatment, (b) ways to get more out of the DTx by using the product as intended, and (c) any number of other communication strategies to give the patient a behavioral cue to move them into a pattern of use that is more likely to lead to greater benefit from the DTx.

Future development of this approach could include moving from a binary classification of whether the patient was using the product as intended to a continuous outcome, for example indicating the total amount of time in each daily task where they were using the product in the intended manner. This could be useful for a number of reasons. First, missions which are currently near the classification boundary and would register as “not played as intended” could instead result in more granular feedback as to the level of engagement as intended (e.g., 73% of a given mission). A continuous output could also serve better as a feature in other models or analyses to examine patterns of usage, effectiveness of different messaging campaigns around proper use of the product, or differences in effectiveness of the DTx for different patients.

A limitation of the current approach is that the machine learning model (a random forest classifier) is moderately complex, and so explaining how the model arrived at a particular decision is not straightforward. Explainable Boosting Machines can be used to create a model that can be as accurate as a random forest while simultaneously providing output that can be easily interpreted (13). We have experimented with these models and found that they produce similar results.

While the specific methods and tooling used for our DTx will not likely transfer directly to another DTx the overall approach could be replicated with similar labeling, feature engineering, and model training efforts. It will require sufficient telemetry recorded, such that an expert observer might discern with high confidence whether or not the data stream represents use as intended. This sort of tool could become a standard feature of DTx, ensuring that products that have undergone such rigorous clinical validation can consistently prove out their benefit in the real world.

## Data Availability

The authors agree to share de-identified labels (“played as intended” or “not played as intended”) and associated classifier predicted labels for each mission included in the training and test datasets following the completion of a Data Use Agreement. Proposals should be directed to medinfo@akiliinteractive.com.
